# Balance and Fall Risk Assessment in Community-Dwelling Older Adults after Recovery from COVID-19: A Cross-Sectional Study

**DOI:** 10.3390/sports11020028

**Published:** 2023-01-28

**Authors:** Amira E. El-Bagalaty, Mariam El-Sayed Mohamed, Osama R. Abdelraouf, Mohamed A. Abdel Ghafar, Alshaimaa K. Abdelaal, Abdelgalil A. Abdelgalil, Gihan S. Mousa

**Affiliations:** 1 Department of Physical Therapy for Pediatric, Faculty of Physical Therapy, Cairo University, Giza 12613, Egypt; 2 Department of Physical Therapy for Cardiovascular/Respiratory Disorders and Geriatrics, Faculty of Physical Therapy, Cairo University, Giza 12613, Egypt; 3 Physical Therapy Program, Batterjee Medical College, Jeddah 21442, Saudi Arabia; 4 Department of Biomechanics, Faculty of Physical Therapy, Cairo University, Giza 12613, Egypt; 5 Department of Physical Therapy for Musculoskeletal Disorders & Its Surgery, Faculty of Physical Therapy, Cairo University, Giza 12613, Egypt; 6 Department of Physical Therapy, Faculty of Applied Medical Sciences, Umm Al-Qura University, Mecca 24382, Saudi Arabia

**Keywords:** coronavirus, older adults, Biodex, Berg Balance Scale

## Abstract

Background: SARS-CoV-2 atypical symptoms in older persons include falls, confusion, dizziness, and unusual weariness. Falls and their consequences are among the most prevalent causes of disability among older adults, significantly lowering quality of life and resulting in the loss of independence as well as impaired psychosocial functioning. The study purpose was to examine the impact of the SARS-CoV-2 infectious disease on balance in community-dwelling older adults. Methods: Sixty-four older adults aged ≥ 60 years from both sexes, 31 treated for SARS-CoV-2 infection and 33 matched normal controls participated in the study. The Biodex Stability System (BSS) and Berg Balance Scale (BBS) were used for evaluation of balance and fall risk. The correlation between the Biodex overall stability index and the Berg Balance Scale score was investigated. Results: When compared to controls, the SARS-CoV-2 group had significantly higher values of the Biodex overall stability index (OSI) (*p* = 0.011), anterior–posterior stability index (APSI) (*p* = 0.013), mediolateral stability index (MLSI) (*p* = 0.018), and fall risk index (FRI) (*p* = 0.008), as well as statistically lower scores on the Berg balance scale (*p* = 0.003). A moderate negative correlation was found between the two assessment tools in the SARS-CoV-2 group. Conclusion: Balance impairment and an increased risk of falling are among the outcomes of SARS-CoV-2 in community-dwelling older adults.

## 1. Introduction

Severe Acute Respiratory Syndrome Coronavirus 2 (SARS-CoV-2) has had a tremendous impact worldwide, causing a high rate of co-morbidities and death [1]. About 83 million cases have been reported, with over 1.8 million deaths [2]. The epidemiological and medical characteristics, pathophysiology, and consequences of the acute phase of SARS-CoV-2 are documented [3,4]. However, some of the illness’s long-term complications are still unknown [2].

Dyspnea, pyrexia, cough, malaise, abdominal pain, diarrhea, and poor appetite are the most common symptoms reported in SARS-CoV-2 cases [5]. However, older patients may experience atypical SARS-CoV-2 symptoms such as delirium, disorientation, dizziness, and physical exhaustion [6]. According to Norman et al., [7] balance impairments including falls are identified among the common presentations of SARS-CoV-2.

Based on the sites of infection, the virus can cause a wide variety of complications in the human systems [8]. Involvement of the central and peripheral nervous systems is also a risk, which can take place directly through nerve tissue invasions or indirectly through inflammatory processes [9]. The virus can enter the central nervous system via the olfactory bulb, producing inflammation and demyelination [10]. Neurological abnormalities might appear not only during the active disease, but also as post-neurological issues following infection recovery [11].

Previous research revealed that SARS-CoV-2 is to blame for a wide range of neurological symptoms, which can be divided into three types. The first path entails the neurological effects of lung disease and accompanying systemic disorders such as systemic inflammatory reactive syndrome. The virus’s direct central nervous system (CNS) invasion causes neurological symptoms in the second pathway. Guillain–Barre syndrome and its variants constitute the third pathway, which includes post-infectious immune-mediated consequences [12].

Balance integration is a vital part of human life that is carried out in the central nervous system using input from the vestibular, visual, and proprioceptive systems [13]. About 30% of the individuals in community-dwelling older populations in low- and middle-income countries might experience falls, with half of them being recurrent fallers [14]. Fall fractures occur in 5 to 10% of all falls, and they account for more than 90% of all hip fractures [15].

The Berg Balance Scale (BBS) is one of the clinical evaluation tools for evaluating balance capability in the older adults. Moreover, the scale has been used in several studies to identify the risk of falling in community-dwelling older persons [16]. However, the test has some flaws, including the inability to assess reactive postural control, and the fact that the Berg Balance Scale focuses far less on gait and the dynamic aspect of balance. As a result, additional tests are required for a full balance assessment, increasing health practitioners’ workload [17].

The Biodex Stability System (BSS) is one of the tools presently being used to assess body control both dynamic and limit of stability. It has been reported that the BSS can assess functional stability, since the size of the base of support on the unstable Biodex platform fluctuates constantly [18]. However, Cho et al. [19] showed that the Biodex Stability System-acquired stability indices represent dynamic stability.

Based on a few case reports and clinical observations, the influence of SARS-CoV-2 infections on balancing mechanisms has been hypothesized [10]. As a direct consequence, dizziness appears to be one of the most frequent balance issues in about one-third of patients [11]. In a retrospective study of 214 patients admitted with COVID-19 in a hospital in Wuhan, 36.4% presented some type of neurological manifestation, which was categorized as CNS involvement (24.8%), peripheral (10.7%) and musculoskeletal (10.7%). Neurological symptoms were more frequent in severe COVID-19 patients (45.5% vs. 30%) [20].

Considering these findings, only questionnaires rather than objective tests have been used to assess balance in SARS-CoV-2 illness [21]. It is critical to document and categorize an individual’s movements in order to determine his or her functional capacity and level of overall of mobility [22]. A 2018 systematic review recommended the use of two assessment tools in combination to increase the diagnostic accuracy of the risk of fall, which might be difficult in older adults because of health issues and limited physical ability [23]. As far as we know, no previous research has investigated the correlation between balance indices derived from Biodex Stability System and Berg Balance Scale to determine if they lead to a similar conclusion or assess different constructs of postural balance. Therefore, the purpose of this research was twofold: first, to investigate the impact of SARS-CoV-2 infection on balance in community-dwelling older adults, and second, to study the correlation between balance assessment using the Biodex Stability System and Berg Balance Scale in both groups.

## 2. Materials and Methods

### 2.1. Study Design

This is an observational cross-sectional study and was conducted during the period April 2021 to November 2021 at the college biomechanical lab. The research was authorized by the Ethics Committee in Batterjee Medical College (Res-2022-0033) in accordance with the Declaration of Helsinki’s ethical standards [24].

### 2.2. Participants

**Figure 1:**
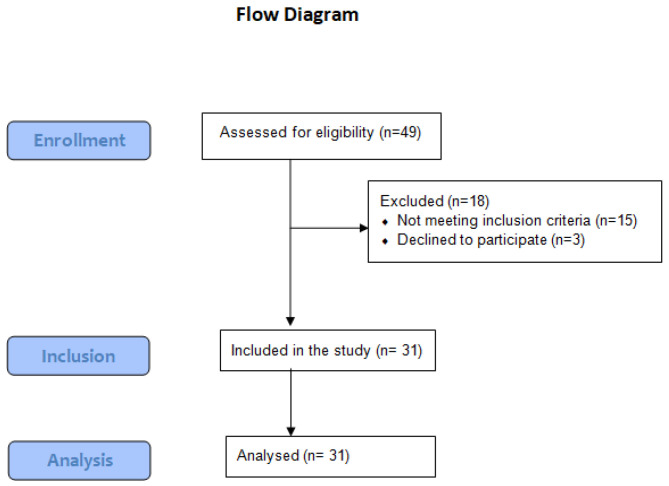
The flow diagram of the of the SARS-CoV-2 group through the stages of the study.

Thirty-one community-dwelling older adults (22 males and 9 females) who recovered from SARS-CoV-2 infection participated in this study. Participants were identified through retrospective review of the medical admission files of five local hospitals. Patients aged 60 years or more [25], diagnosed with mild or moderate SARS-CoV-2 infection [26], and discharged in the last six months before April 1st were considered eligible. Recovery was confirmed after two consecutive negative real-time reverse polymerase chain reaction tests. The average length of stay in the hospital in this group was 21.8 ± 7.6 days. Out of the forty-nine patients who were initially contacted, thirty-one participated in the study. This group was matched based on demographic data and functional capacity level with thirty-three older adults (23 males and 10 females) with no previous history of various signs and symptoms or positive testing for SARS-CoV-2 infection as confirmed by participants and/or family members. These controls were recruited through announcements in local senior citizens community centers. Individuals were excluded from the study if they were unable to stand or walk independently for at least 10 m [27] without assistive devices or needed help with ADLs; had a history of falling that caused musculoskeletal trauma; had received prior balance exercises or physical therapy; had a history of systemic or neurological diseases that might affect balance; or were unable to comprehend and follow verbal instructions. Figure 1 shows a flow diagram of the subjects’ selection. The study goal and procedures were explained to all participants, and they completed an informed consent form. The sample size was calculated to be 29 participants in each group using G*power 3.1 software (Universities, Dusseldorf, Germany) [28] based on α = 0.05, power = 0.80, and an effect size = 0.85.

### 2.3. Procedures

#### 2.3.1. Evaluation Procedures

The Biodex Stability System (BSS, Biodex Inc., Shirley, NY, USA) was used to assess the participants’ capability to sustain dynamic postural stability when exposed to a dynamic load. It is composed of a circular platform that is free to move in the anterior–posterior and medial–lateral axes simultaneously, with the ability to control the movement degree of the platform with 12 levels. The Biodex Stability System device is interfaced with dedicated software (Biodex, Version 1.08), allowing the Biodex Stability System to measure the degree of tilt in each axis, thus providing an average sway score. Eight springs located underneath the outer edge of the platform provide the resistance to movement (stability level of the platform). Resistance levels range from 8 (most stable) to 1 (least stable). The postural stability test (PST) was used to measure the overall stability index (OSI), anterior–posterior stability index (APSI), mediolateral stability index (MLSI) and fall risk index (FRI). Low scores on these tests indicated that the individual was stable, whereas high scores indicated a lack of balance and a higher risk of falling. Patients stood barefoot on the platform with their eyes open. Before the test, the subjects were provided 3 min of Biodex Stability System training prior to the test to acquaint themselves with the apparatus. Each assessment lasted 20 s, with a 10 s break in between. For each mode, the average of the three trials was calculated for each individual. 

The Berg Balance Scale (BBS) consists of the following 14 items related to balance-specific activities, ranging from sit-to-stand to standing on one leg: (1) sitting unsupported, (2) standing unsupported, (3) standing with eyes closed, (4) standing with feet together, (5) standing on one foot, (6) turning to look behind, (7) retrieving an object from the floor, (8) tandem standing, (9) reaching forward with an outstretched arm, (10) sitting to standing, (11) standing to sitting, (12) transfer, (13) turning 360°, and (14) stool stepping. All these items must be completed safely. Each item on the Berg Balance Scale (14 items) is scored on a five-point Likert scale (0–4) in which 0 (unable to try or need assistance to prevent falling) and 4 (able to lift the leg independently and hold for >10 s), resulting in a maximum possible score of 56 (lower points score means high possibility for balance loss and higher points score means superior performance). Most items require participants to keep their balance while attempting the tasks. The participant is free to choose which leg to stand on or how far to reach. A stopwatch to evaluate stance and sit performance time under different conditions, a ruler (2, 5, and 10 inches) to measure forward limit of stability, two chairs with reasonable height (one with and one without an armrest) for a sit-to-stand transition activity, and a stepper are all needed to complete the scale testing. Full description of the test procedures were reported in the literature [29,30].

#### 2.3.2. Statistical Analysis

The Statistical Package for Social Sciences (SPSS) for Windows version 20.0 (SPSS, Armonk, NY, USA, IBM Corp.) was used in this study for data analysis. Normal distribution and homogeneity of the data was checked prior to conduction of data analysis using Shapiro–Wilk and Levene’s tests (*p* > 0.05). Chi-square test was used to determine the difference in gender, number of different health conditions, and socioeconomic factors in both groups. Independent t-test was used to determine the difference in age, height, weight, BMI, Biodex stability indices, and Berg Balance Scale score between both groups. Then, Pearson’s product moment correlations were used to examine relationships between balance overall and Berg Balance Scale scores. The strength of the relationships was described as detailed by Portney, where 0.00–0.25 indicated little or no relationship; 0.26–0.50 indicated fair degree of relationship; 0.51–0.75 indicated moderate-to-good relationship; and 0.76–1.00 indicated good-to-excellent relationship. The significance level of a *p*-value of ≤0.05 was considered statistically significant using 95% confidence intervals [31].

## 3. Results

There was no significant difference between groups in age, weight, height, BMI, socioeconomic factors, the number of participants with history of falls, and diabetes mellitus (*p* > 0.05). Only significant differences were found between the number of participants with history of hypertension and cardiopathies in both groups (*p* < 0.05), as shown in Table 1. 

The result of the independent t-test indicated a substantial difference regarding all the measured Biodex Balance indices and the Berg Balance Scale score between the SARS-CoV-2 group and the control group (*p* < 0.05). The SARS-CoV-2 group had significantly higher values of anterior–posterior stability index (*p* = 0.013), mediolateral stability index (*p* = 0.018), overall stability index (*p* = 0.011), and risk of fall index (*p* = 0.008) when compared with the healthy control group. Furthermore, the Berg Balance Scale scores were statistically greater for the control group than those who got infected with SARS-CoV-2 virus (*p* = 0.003). Measured variables are presented in Table 2.

Pearson correlation coefficient test showed moderate negative correlations between Berg Balance Scale score and Biodex overall stability index (*p* = 0.003 * and r = −0. 58) and between Berg Balance Scale score and Biodex fall risk index (r = −0.65) in the SARS-CoV-2 group. On the other hand, fair negative correlations between Berg Balance Scale score and Biodex overall stability index (*p* = 0.001 * and r = −0.39) and between the Berg Balance Scale score and Biodex fall risk index (r = −0.47) were found in the control group.

## 4. Discussion

The objective of this research was to study the impact of SARS-CoV-2 infection on balance in community-dwelling older persons, as well as the correlation between Biodex Stability System and Berg Balance Scale balance assessments. The present study’s findings revealed impaired balance and increased fall risks in community-dwelling seniors after recovery from SARS-CoV-2 infection compared with controls as measured by Biodex Stability System and Berg Balance Scale.

The results of this study can be explained by the fact that SARS-CoV-2 could affect the neuronal tissue either by a direct infection of the central nervous system (CNS) through the olfactory bulb or through related vascular injury as vasculitis or vasculopathy [32]. Post-infectious immune-mediated consequences is a third pathway through which the virus affects the CNS [12]. These big neural changes might affect the CNS ability to respond efficiently to visual, vestibular, and proprioceptive feedback about body posture.

Yılmaz et al. [33] also reported that instead of causing vertigo, COVID-19 illness can produce dizziness which could be owing to vestibular and visual system involvement, or their central linkages. Sequelae in the balance-related systems may persist for months, as they have lasted even after the patients’ recovery. Furthermore, Mao et al. [20] evidenced that SARS-CoV-2 can infect the neurological system, skeletal muscle, and respiratory tract, either directly or indirectly through inflammatory reactions.

Dizziness and consequent imbalance are well-recognized problems among older adults [34]. Mustafa and Taya [35] reported that SARS-CoV-2 infections demonstrated a significant impact on saccular vestibular hair cell functioning that may cause many vestibular illnesses such as vestibular neuritis, benign paroxysmal positional vertigo, and orthostatic dysfunction. Inner ear components are especially vulnerable to ischemia due to their high energy demand, which can lead to balance disturbances [36].

An important point to be considered is that side-effect symptoms in patients with SARS-CoV-2, such as muscular fatigue, muscle aches, tingling sensations, and reactive arthritis, can affect any joint, but they occur more frequently in lower limbs [37]. Huang et al. [2] found that fatigue and muscle weakness were prevalent for six months in adult patients discharged from the hospital after recovery from SARS-CoV-2. 

From another perspective, balance impairment in the SARS-CoV-2 group could be attributed to their hospital stay, with an average length of 21.8 days. The finding of negative hospitalization outcomes in older adults was described as early as the 1990s. Margitić et al. [38] reported that among 1279 patients in acute medical wards of five hospitals in the United States, 42% experienced functional decline in Activities of Daily Living (ADL), compared to two weeks prior to hospitalization. Only half of those who experienced post-hospitalization functional decline returned to their basic functioning at four months after discharge. More recent studies support these early findings, showing that patients aged 65 and older often suffer from functional decline during and after hospitalization, and functional decline has been shown to be sustained up to one year following discharge [39,40].

The results of this study demonstrated a negative correlation in both groups between Biodex OSI and the Berg Balance Scale values. However, the correlation was moderate in the post-COVID-19 group, and fair for the control group. Consequently, the Biodex Stability System is highly recommended to identify fall risks in older adults who recovered from SARS-CoV-2 in order to avoid the flaws of Berg Balance Scale. This correlation can be attributed to a decline in activity associated with poor physical function that decreases the gap between static and dynamic balance assessments in older adults. Only a few older adults followed the WHO guidelines on physical activity and sedentary behavior that they should do at least 150–300 min of moderate-intensity aerobic physical activity throughout the week, for substantial health benefits [40].

The SARS-CoV-2 pandemic and consequent lock-down orders have resulted in a decline in physical functioning in countries all over the world [41]. Sedentary behavior caused by stay-at-home restrictions altered normal muscular activity during daily mobility, which can lead to muscle atrophy and other motor function problems in community-dwelling older persons [42]. Consistently with these findings, Parsa et al. [43] have revealed moderate negative correlation between the stability indices of the Biodex Stability System and Berg Balance Scale scores in stroke individuals. Murphy and Roberts [44] also reported that positional static stability on the force platform and the Berg Balance Scale scores in geriatric patients with a cerebrovascular accident had a strong correlation. Many researchers have studied balance in patients with multiple sclerosis, Parkinson’s illness, and a history of falling. The Biodex OSI and Berg Balance Scale scores had a substantial correlation in all these investigations [45,46,47,48].

Contrary to the results of this study, several investigations evaluated positional perturbation on all sides and found a non-significant correlation between laboratory static stability testing and Berg Balance Scale scores, which also measure functional balance [18,49]. The different types of tested stability, assessment methodology, and evaluated sample might explain the discrepancies in the current study conclusions.

This study has some limitations; first, although G* power software was used to determine the minimum number of subjects for adequate study power, a bigger sample size would increase the strength of the study results. Second, the inability to hear or comprehend verbal instructions was one of the study exclusion criteria; however, lab auditory and hearing assessment was not performed. Finally, there were also no available data about the medications taken by each group that might affect balance.

## 5. Conclusions

The SARS-CoV-2 infection was found to be one of the multifactors that affect postural balance in community-dwelling older adults. This balance decline seems to persist even after recovery from the disease. Moreover, it is recommended to use the Biodex Stability System in assessments of balance in this specific population to avoid different flaws of the Berg Balance Scale.

## Figures and Tables

**Table 1:** The demographic data of both groups.

Groups	SARS-CoV-2 Group n = 31	Control Group n = 33	*p*-Value
	Mean ± SD	Mean ± SD	
Age (years)	69.79 ± 3.21	70.89 ± 4.63	0.542 ^a^
Height (m)	1.62 ± 0.12	1.65 ± 0.17	0.680 ^a^
Weight (kg)	75.39 ± 9.38	77.54 ± 10.27	0.286 ^a^
BMI	28.67 ± 2.67	30.13± 5.54	0.135 ^a^
Gender distribution (Male/Female)	(22/9)	(23/10)	0.796 ^b^
Health conditions			
Hypertension	25/31	22/33	0.045 * ^b^
Cardiopathies	24/31	20/33	0.041 * ^b^
Diabetes	17/31	19/33	0.634 ^b^
Falls	9/31	10/33	0.721 ^b^
Socioeconomic Factors			
Living at home with family	25/31	24/33	0.673 ^b^
Living at home alone	6/31	9/33	0.598 ^b^
Smoking	12/31	13/33	0.726 ^b^

Data are illustrated as mean ± standard deviation; ^a^ refers to independent *t*-test; ^b^ refers to Chi-square test; * *p* value > 0.05 means statistically significant difference.

**Table 2:** Comparison between Biodex Stability indices and Berg Balance Scale score between the SARS-CoV-2 group and the control group.

Groups		SARS-CoV-2 Group n = 31 Mean ± SD	Control Group n = 33 Mean ± SD	*p*-Value
Biodex Balance indices	APSI	3.05 ± 0.42	2.26 ± 0.31	0.013 *
	MLSI	2.17 ± 0.28	1.43 ± 0.2	0.018 *
	OSI	3.25 ± 0.45	2.16 ± 0.32	0.011 *
	FRI	5.32 ± 0.79	3.86 ± 0.46	0.008 *
Berg Balance Scale score		41.92 ± 5.64	50.02 ± 4.64	0.003 *

Data are illustrated as mean ± standard deviation. APSI, anterior–posterior stability index; MLSI, mediolateral stability index; OSI, overall stability index; FRI, fall risk index, * *p* value < 0.05 means statistically significant difference.

## Data Availability

The materials that support this manuscript are available from the corresponding author upon reasonable request.
